# Strengthening health systems resilience using environmental surveillance for COVID-19 and antimicrobial resistance in the Philippines

**DOI:** 10.5365/wpsar.2022.13.2.930

**Published:** 2022-06-23

**Authors:** Miguel Antonio Salazar, Leslie Michelle M. Dalmacio, Aileen H. Orbecido, Ruth C. Abanador, Michael Angelo Promentilla, Arnel B. Beltran, Renan Ma. T. Tanhueco, Marilen Parungao Balolong

**Affiliations:** a Division of Data, Strategy & Innovation Team, World Health Organization Regional Office for the Western Pacific, Manila, Philippines.; b Department of Statistics, University of California, Los Angeles, CA, United States of America.; c Division of Health Security and Emergencies, World Health Organization Regional Office for the Western Pacific, Manila, Philippines.

As an early warning strategy for coronavirus disease 2019 (COVID-19), environmental detection of severe acute respiratory syndrome coronavirus 2 (SARS-CoV-2) in wastewater was integrated into pandemic responses in Australia, Germany, New Zealand, the United Kingdom of Great Britain and Northern Ireland, and the United States of America. (*1*-*3*) Research on methodologies for wastewater surveillance (WWS) for SARS-CoV-2 has been undertaken in Ecuador, France, India, Israel, Italy, Japan, the Netherlands, Spain and Turkey, to name a few. (*1*) WWS of SARS-CoV-2 has been demonstrated as an early warning system for outbreaks of COVID-19 (*3*) and could be a useful tool for communities across the Philippines.

Antimicrobial resistance (AMR) is a pervasive global health concern, complicating treatment of infectious diseases and routine medical procedures. The Philippines has experienced increasing numbers of infections resistant to specific antibiotic combinations, for example, carbapenem resistance in *A. baumannii* has increased from below 30% in 2009 to 56% in 2017. (*4*) Globally, reservoirs of antimicrobial-resistant genes (ARGs) have been found in wastewater. This is of interest to health-system managers as ARGs found in wastewater treatment plants have been shown to follow patterns of resistance in clinical isolates. (*5*)

New molecular diagnostic laboratories established for the detection of SARS-CoV-2 in the Philippines could be used for other emerging infectious diseases and, thus, contribute to improved resilience during future epidemics. This article discusses how the strengthened monitoring and surveillance capacity developed for SARS-CoV-2 in the Philippines provides opportunities for environmental surveillance of emerging infectious diseases and AMR.

Environmental surveillance, specifically WWS, could be used as an adjunct to clinical and laboratory diagnosis of individuals for the detection of local outbreaks. Similar to clinical testing, WWS utilizes reverse transcription-quantitative polymerase chain reaction (RT-qPCR). (*1*, *2*) Community outbreaks of COVID-19 could, therefore, be detected using WWS regardless of the local government’s capacity for clinical testing. As SARS-CoV-2 can be shed through stool, WWS can detect possible outbreaks from symptomatic and asymptomatic members of the population even before community cases or hospital admissions are reported. Targeted testing of communities guided by WWS reduces the need to test larger populations, thus reducing the cost to governments. (*2*)

**Figure 1:**
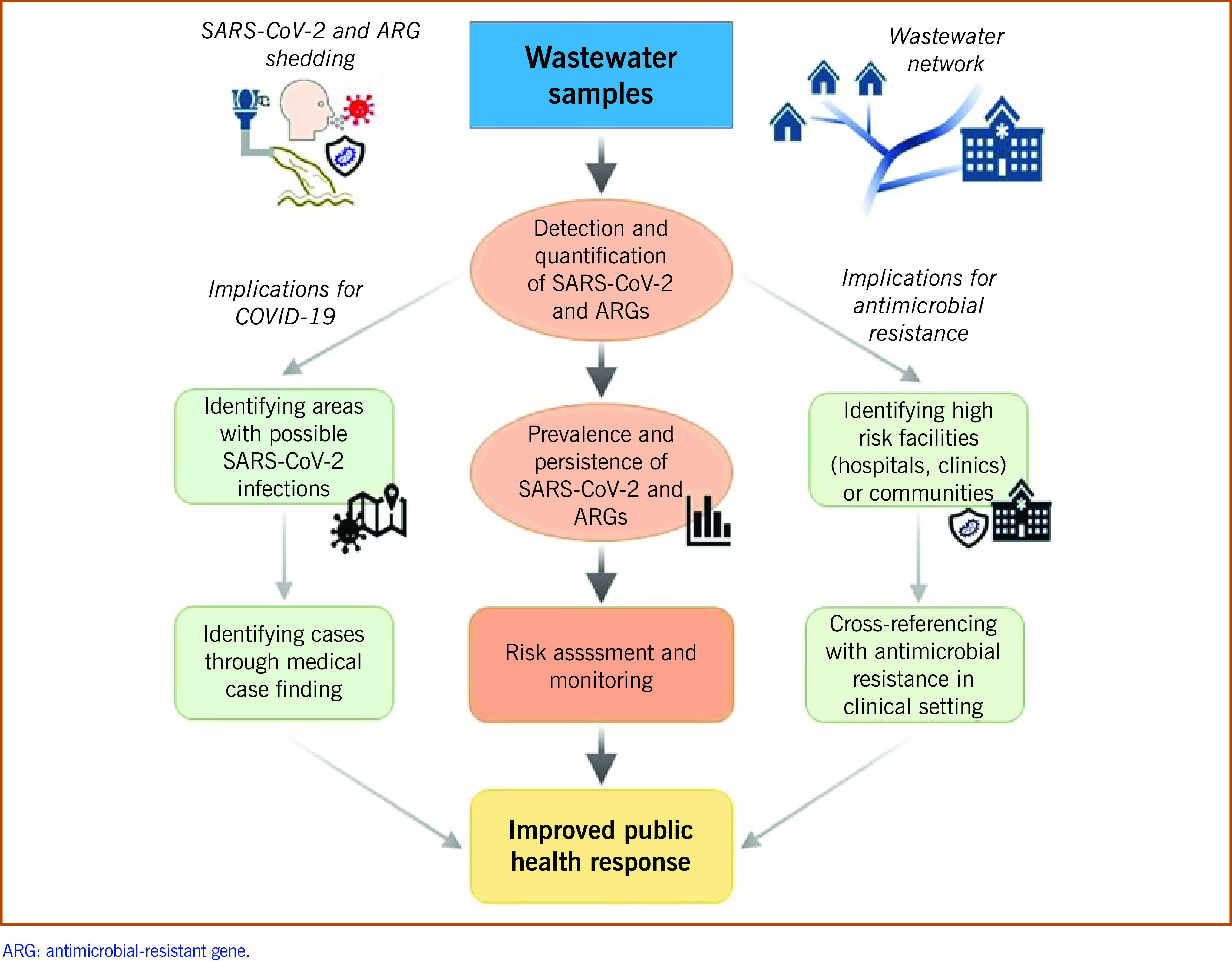
Implications of environmental surveillance using wastewater for COVID-19 and antimicrobial resistance

It is possible for WWS to detect and analyse chemical or biological compounds to understand the health status of communities. (*1*, *2*) Public health and research teams have previously used environmental surveillance for antimicrobial-resistant organisms, ARGs (*5*) and the Global Polio Eradication Initiative. (*6*) Detection and quantification of SARS-CoV-2 and ARGs in wastewater provides a risk assessment opportunity for the identification of communities at risk of COVID-19 outbreaks and AMR patterns from hotspots such as medical facilities. This practice could provide early warning to health authorities and increase case-finding efforts for COVID-19 in targeted communities. It could also improve infection prevention and control in AMR-affected medical facilities (**Fig. 1**).

[**insert** Figure 1]

The Philippines has recently increased its molecular laboratory testing capacity for SARS-CoV-2. The number of public and private sector laboratories with the ability to detect SARS-CoV-2 using polymerase chain reaction (PCR) increased from one in the first quarter of 2020 to 247 by the end of April 2022. (*7*) Prior to the pandemic, capacity in molecular diagnosis for infectious disease was tasked to the Research Institute for Tropical Medicine (RITM), which hosts the Antimicrobial Resistance Surveillance Program with 24 sentinel sites. RITM has also been developing capacity in whole-genome sequencing of AMR. (*4*)

Compared to human clinical surveillance in the Philippines, AMR surveillance for animal and environmental health is still under development. There have been few studies carried out on the detection of AMR residues in agriculture and food animal production settings. (*8*) RITM has been strengthening its environmental surveillance capacity through its polio WWS, while select academic institutions in the country are focusing on AMR detection in wastewater and agriculture. (*4*, *8*, *9*) Currently there is no national initiative for SARS-CoV-2 WWS; however, there are research initiatives underway on the topic.

Environmental surveillance must involve multisectoral support from biology, chemistry, clinical medicine, chemical engineering, civil engineering, epidemiology, microbiology, public health and waste management. Such a multidisciplinary support team would ensure a thorough understanding of wastewater infrastructure and microbiological detection methods and its integration into pandemic response and health systems. As the Philippine agency assigned to health protection, the Department of Health (DOH), with RITM, can develop, implement and study the cost–effectiveness of multidisciplinary WWS in the response to emerging infectious diseases.

Bringing Filipino institutions and multidisciplinary professionals together with experience in environmental and clinical surveillance could be a starting point for a national One Health integrated surveillance system where clinical and environmental samples from human health systems, animal health systems, food systems and the environment can be analysed and correlated. This system could inform hygiene, sanitation and infection prevention strategies to reduce the risk of spreading AMR and infectious disease outbreaks of pandemic potential. A One Health integrated surveillance system could increase its reach by building on the growing capacity of Filipino medical technologists in the use of RT-qPCR as they handle more samples due to the surge of COVID-19 cases. Capacity building could also be supplemented by online training from experienced global researchers through today’s communication technology. The Philippine Interagency Committee on Antimicrobial Resistance (ICAMR), established in 2014 and comprising the DOH, Department of Agriculture, Department of Science and Technology, Department of Interior and Local Government, and Department of Trade and Industry, could oversee the One Health integrated surveillance system, as the committee is already strengthening surveillance and laboratory capacity for AMR. (*10*)

As epidemics continue to affect the Philippines, improved preparedness, response and resilience to emerging infectious diseases including AMR could be supplemented by a One Health integrated surveillance system. To implement such a system, the country would need to develop capacity in environmental surveillance, including WWS, making use of existing infrastructure and expertise while exploring possibilities for collaboration with global experts and international partners. National agencies and committees, such as the DOH, RITM and ICAMR, would have to take on responsibility for overseeing and leading this initiative. A multidisciplinary approach and the identification of relevant Philippine institutional partners would be needed to sustain this initiative and prepare for emerging infectious diseases and chronic health-system challenges.
